# MALAT1 Long Non-coding RNA and Its Role in Breast Carcinogenesis

**DOI:** 10.32607/actanaturae.11905

**Published:** 2023

**Authors:** M. M. Tsyganov, M. K. Ibragimova

**Affiliations:** Cancer Research Institute, Tomsk National Research Medical Center, Russian Academy of Sciences, Tomsk, 634050 Russian Federation; Siberian State Medical University, Tomsk, 634050 Russian Federation; National Research Tomsk State University, Tomsk, 634050 Russian Federation

**Keywords:** MALAT1, NEAT2, breast cancer, long non-coding RNAs, carcinogenesis

## Abstract

Our genome consists not only of protein-coding DNA, but also of the non-coding part that plays a very important role in the regulation of all cellular processes. A part of the non-coding genome comes with non-coding RNAs (ncRNAs), and disruption of the functional activity of these RNAs may be associated with oncogenesis in various cancer types. There exist two types of ncRNAs: small and long non-coding RNAs, which are classified according to their transcript length. Long non-coding metastasis-associated lung adenocarcinoma transcript 1, *MALAT1 *RNA (*NEAT2*), is a long non-coding RNA of particular interest. The aforementioned transcript takes part in the regulation of numerous cellular processes and pathogenesis of different malignant tumors, including breast tumors. This review focuses on experimental and clinical studies into the role of MALAT1 in carcinogenesis and the progression of breast cancer.

## INTRODUCTION

Breast cancer (BC) remains one of the most common malignant tumors affecting women [1]. Breast cancer is highly heterogeneous, which makes it different in how sensitive it is to therapy, its prognosis and risk of metastatic spread and recurrence, thus, reducing treatment effectiveness. Therefore, personalized preoperative therapy moves to the forefront in breast cancer patients [2]. Molecular markers for breast cancer such as tumor cell membrane receptors, the p53 protein, antigen Ki-67, the *BRCA1 *and *BRCA2 *genes, various microRNAs, etc. are currently well-understood, which allows one to classify tumors and predict treatment outcome [3]. Five molecular biological subtypes of breast cancer are recognized today: ER+ luminal A breast cancer (HER2-negative, low Ki-67 expression (≤ 20%), and high progesterone receptor (PR) level (≥ 20%)); HER2-negative luminal B breast cancer: ER+, HER2-, one of the following factors is present: high Ki-67 expression (≥ 30%) or low PR level ( < 20%); HER2-positive luminal B breast cancer: ER-positive, HER2-positive, any level of Ki-67 expression, any PR level; HER2+: HER2+, ER- and PR-, any level of Ki-67 expression; and triple negative breast cancer (TNBC): ER-, PR-, HER2- [4]. However, almost no target is effective in triple-negative breast cancer.

The advances in genome sequencing technology have revealed that, along with protein-coding RNAs, the human genome encodes nontranslating (non-coding) RNAs (ncRNAs) constituting most of the genome (~ 98%) [5]. Non-coding RNAs are involved in genetic and epigenetic regulation; therefore, their functions and participation in tumor progression are being currently vigorously studied [6]. ncRNAs are subdivided into small (micro-) and long non-coding RNAs (miRNAs and lncRNAs, respectively). Long non-coding RNAs, which perform many different functions in the cell and take part in various processes, are of particular interest [6, 7]. The functions of 2% of lncRNAs have been identified thus far. There are three categories of functions performed by lncRNAs. They act as signaling molecules, regulate transcription by participating in the assembly of RNA polymerases in the enhancer domain, initiate RNA cleavage, and are associated with pluripotency and cellular reprogramming. lncRNAs act as miRNA traps or guides binding proteins and delivering them to the regions where they become involved in the *trans*- and *cis*-regulation of gene expression by binding to DNA:RNA heteroduplexes or RNA:DNA:DNA triplexes and interact with Polycomb group and Trithorax group proteins, thus preventing them from performing histone modification and exerting any degree of epigenetic regulation and chromatin remodeling. lncRNAs initiate the assembly of the RNA complexes that act as protein assembly sites and control the protein function under stress conditions [6, 8, 9, 10, 11, 12]. MALAT1 RNA associated with metastatic lung adenocarcinoma is one of the interesting lncRNAs [13].

## MALAT1 LONG NON-CODING RNA

MALAT1 RNA was first discovered when studying gene expression in metastatic non-small cell lung cancer (NSCLC) [13]. MALAT1, also known as NEAT2 (nuclear-enriched abundant transcript 2), resides in the nucleoplasm in nuclear speckles (structures performing various functions, the main one being the regulation of pre-mRNA splicing and transcription) [14]. The intronless *MALAT1 *gene localized in the 11q13.1 locus encodes the ~ 8700-nt-long transcript [15, 16]. The *MALAT1 *gene is located in a region characterized by a high density of genes with very high synthetic evolutionary conservation [17]. Thus, a unique feature of *MALAT1 *is that its nucleotide sequence is conserved (in vertebrates, the overall conservation of the 3’-terminal sequence is > 50% and 80%) [18]. The MALAT1 transcript usually has a long half-life: it remains stable for 16 h in human B cells and for 9–12 h in tumor cells [19]. The half-life of MALAT1 is longer than that of other lncRNAs, probably because of the triple helical structure present on its 3’-end [20].

**Fig. 1:**
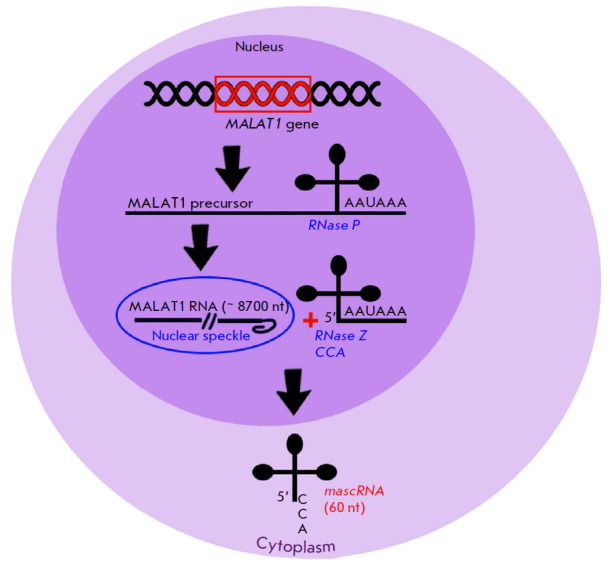
Scheme of the synthesis of MALAT1 long non-coding RNA in a cell

*MALAT1 *is transcribed by RNA polymerase II from the long arm of human chromosome 11 (11q13) (*Fig. 1*). Formation of this lncRNA depends on tRNA processing that produces two non-coding RNAs from the same locus, which reside in different subcellular compartments and perform different functions [21]. The RNase P endonuclease recognizes this tRNA-like structure and cleaves it to simultaneously generate the mature 3’-end of the MALAT1 long transcript and the 5’-end of the tRNA-like small RNA. Additional enzymes, which are involved in tRNA biogenesis, including RNase Z and the CCA-adding enzyme, then process small RNA to form the 61-nt-long mature transcript known as mascRNA (*MALAT1*-associated small cytoplasmic RNA). Once the MALAT1 primary transcript is processed, mascRNA is exported to the cytoplasm while the long transcript remains in the nucleus in the form of nuclear speckles [22].

MALAT1 long non-coding RNA accumulates in the nucleus, where it plays a crucial role in cancer progression and the formation of nuclear paraspeckles.

**Fig. 2:**
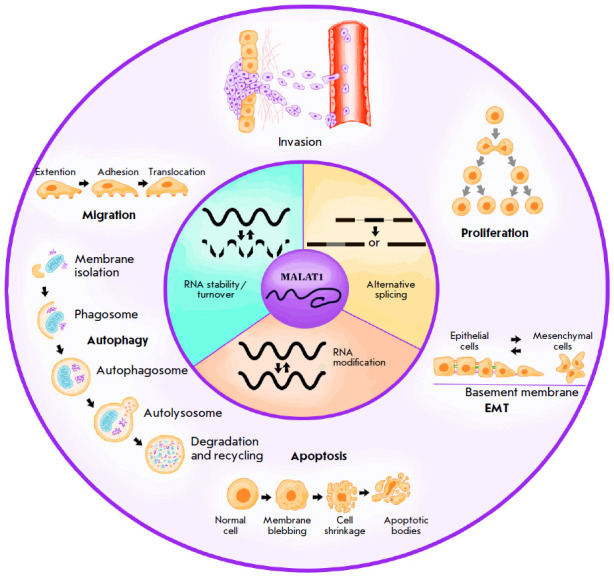
The main functions of MALAT1 long non-coding RNA in a cell

MALAT1 has many different functions (*Fig. 2*): it (1) acts as a nuclear scaffold at the speckle periphery for trans-acting protein factors such as SR proteins, leading to the modulation of pre-splicing and alternative splicing; (2) is involved in the post-transcriptional regulation of the genes associated with cellular motility [23]; and (3) participates in the regulation of many processes, together with microRNAs [24, 25, 26, 27], as well as in epigenetic regulation; e.g., MALAT1 binds to the promoter of the *EEF1A1 *gene encoding eukaryotic translation elongation factor 1 alpha 1 (EEF1A1), resulting in the methylation of histone H3 [28].

Not only does MALAT1 play a regulatory role, but it also participates in various signaling pathways (e.g., in the TGF-β/Smad and p53 pathways) [11, 29]. Interestingly, MALAT1 can bind to other ncRNAs and pre-mRNAs, mostly exclusively through mediator proteins, and bind to chromatin exclusively within the region of actively spliced genes [14].

Alternative splicing is also worth mentioning: changes to it are increasingly often recognized as a potential pathogenic mechanism of carcinogenesis. Alternative splicing is the post-transcriptional mechanism that enhances the transcriptome complexity by expressing many of the different mRNAs of individual genes, thus potentially generating different protein isoforms [30]. The screening of the Database of Expressed Sequence Tags (dbEST) undertaken by Meseure et al. discovered Δsv-MALAT1 (the small variant MALAT1 transcript), which was the main product of the alternative splicing of MALAT1. Breast tumors are mostly characterized by low Δsv-MALAT1 expression levels [31].

## THE ROLE OF MALAT1 IN BREAST CARCINOGENESIS

It is exciting to study the role played by MALAT1 in carcinogenesis, because this RNA is involved in the regulation of numerous cellular processes. Thus, expression of the *MALAT1 *transcript is unstable in patients with different types of cancer and in tumors of different localizations [32]. MALAT1 was first found to be involved in carcinogenesis in patients with nonsmall cell lung cancer and was shown to be associated with a higher risk of metastatic disease and unfavorable outcome in patients with squamous cell cancer and adenocarcinoma of the lung [13]. Weber et al. [33] suggested that the serum level of MALAT1 in lung cancer patients could be a potential biomarker for this disease. Moreover, MALAT1 overexpression is observed in human hepatocellular carcinoma, breast cancer, pancreatic cancer, and colorectal cancer cells [32]; it is also involved in the expression regulation of some genes associated with metastatic ability [10, 34] and tumor progression in breast cancer patients [27]. According to Liu et al. [35], MALAT1 expression is positively correlated with metastatic lung cancer and negatively correlated with disease prognosis; it is an important prognostic marker for patients with NSCLC. The data on MALAT1 involvement in tumor processes have aroused a keen interest in studying the oncogenic role of MALAT1 and its involvement in metastatic breast tumor. Thus, it has been established that MALAT1 plays a critically important role in the regulation of transcription and the cell cycle, epigenetic regulation, as well as in the inflammation and metastatic processes in tumor (*Fig. 2*) [36]. MALAT1 affects the initiation and progression of tumors of various localizations, including laryngeal cancer, cancer of the laryngopharynx, as well as thyroid, esophageal, lung, liver, and ovarian cancers [37, 38, 39, 40]. Therefore, MALAT1 is among the key factors contributing to the regulation of the molecular pathways that lead to phenotypic manifestations of cancer [16]. Below, the role of MALAT1 in breast cancer will be discussed in more detail.

***In vitro *studies**

The mechanisms causing cell migration and invasion and research into the metastatic cascade in breast cancer patients are of significant interest. Many studies have confirmed that MALAT1 is involved in the regulation of cells’ migration and invasion ability. MALAT1 was previously reported to regulate the proliferation of cervical and gastric cancer cells, as well as their cisplatin resistance through the PI3K/Akt pathway [41, 42]. The epithelial–mesenchymal transition (EMT) of tumor cells is one of the first steps in metastatic spread [43]. Xu et al. [23] studied the role of MALAT1 in EMT in breast cancer patients and found that MALAT1 promotes *in vitro *migration and invasion of breast cancer cells (MDA-MB-231, MDA-MB-453, MCF10A, SK-BR-3, and BT549); the lower MALAT1 expression level is associated with metastatic breast cancer; i.e., MALAT1 acts as an EMT inducer by activating the PI3K-Akt pathway [23]. Similar findings were also made by Wu et al. [44], who demonstrated that the PI3K/Akt pathway mediating FOXO1 binding to the *MALAT1 *promoter can be the mechanism through which *MALAT1 *induces EMT and reduces the trastuzumab sensitivity of HER2+ breast cancer cells [44]. A distinctive feature of this study was that MALAT1 expression was evaluated in seven breast cancer cell lines that included cell lines of the ER+/HER2-, ER+/HER2+, ER-/HER2+, and TNBC subtypes. Among these cell lines, the highest MALAT1 expression level was detected in metastatic triple-negative breast cancer cells and trastuzumab- resistant HER2+ cells [44]. Cell cultures of triple-negative breast cancer (MDA-MB-231, primary TNBC cells, Hs578T, and HCC1806) were characterized by lower MALAT1 levels compared to ER+ cells (MCF-7, primary ER+ tumor cells, and T-47D) [27, 45, 46]. It was found that metastasis-associated MALAT1 overexpression can be negatively correlated with the expression of the Nisch product, a tumor suppressor protein whose expression is downregulated in breast cancer patients [47]. In 231-GFP-Nisch cell cultures (MDA-MB-231 with Nisch overexpression), Nisch expression levels are associated with the MALAT1 expression levels: knockout of the *Nisch *gene transcript in these cells increases their proliferation and migration [47].

Zhang et al. [48] showed that tumor cells secrete MALAT1 in recipient cells in order to regulate the proliferation of receptor cells in the tumor microenvironment. MALAT1 expression levels in breast cancer cells are significant: MDA-MB-231 exosomes substantially increase the proliferation of MDA-MB-231 and ZR-75-1 cells; however, exosomes from MDA-MB-231 cells treated with MALAT1-siRNA (small interfering RNA or siRNA targeting MALAT1) reduce cell proliferation in breast cancer patients. Earlier, Jin et al. [45] showed that *MALAT1 *in TNBC cells suppresses cells’ proliferation and invasion ability and triggers apoptosis, which is achieved through reverse regulation of the miR-1 RNA transcriptome and its target protein promoting epithelial–mesenchymal transition, Slug [45]. The mechanism of functioning of this process has been described more thoroughly for the MDA-MB-231 and MCF-7 cultures, using the plasmid transfection method. MALAT1 overexpression enhances cells’ migration and invasion ability by binding to miR-1 and reducing the level of Cdc42, the protein involved in EMT [26]. Furthermore, it was revealed using siMALAT1-mediated inhibition of MALAT1 and, conversely, by inserting the MALAT1 overexpression vector into a breast cancer cell line that MALAT1 expression directly affects the expression of miR-124, a microRNA associated with the suppression of breast cancer progression and that MALAT1 overexpression suppresses the inhibitory effect of miR-124 on breast tumor growth, thus increasing its size [25].

For the culture of 4T14T1 cells, the highly metastatic breast cancer cell line derived from spontaneous mammary tumor in BALB/c mice, Li et al. [49] discovered a new mechanism through which MALAT1 could participate in the regulation of EMT in mammary tumors. The transcript was shown to exhibit pro-inflammatory activity and be able to regulate the lipopolysaccharide-induced inflammation and cellular EMT. An antisense transcript of the *MALAT1* gene, transcribed from the opposite strand and named TALAM1, was also discovered [50]. Having conducted their own study based on this discovery, Gomes et al. showed that overexpression of these transcripts is typical of breast cancer cell lines and that there exists a positive correlation between their expression levels in the studied cell lines. MALAT1 and TALAM1 work together: TALAM1 mediates MALAT1 activity in the presence of TGF-β cytokine [51], a well-known EMT inducer. Nevertheless, it is rather difficult to assess the effect of MALAT1 on cells’ metastatic ability, since different authors have provided different descriptions of this mechanism. The main reasons for the lack of consistency in the data on MALAT1 activity are still to be identified. Differences in the results obtained for tumor cell cultures can probably be assigned to the features of protein expression in different types of cells, as well as to the fact that the MALAT1 transcript forms complexes with different proteins, thus causing opposite effects [47]. Other plausible explanations include the use of cell lines having different genetic backgrounds or differences in culture conditions.

It is notable that the effect of MALAT1 on cell function was uncovered in studies using the A549 lung cancer cell line. A549 cells were transfected with MALAT1 siRNA1 and MALAT1 siRNA2; the control cells were transfected with control siRNA1 and siRNA2, respectively. *MALAT1 *knockdown by siRNA reduced MALAT1 levels by 70–80%, which significantly affected cell motility (this parameter decreased compared to that in the cells transfected with control siRNA). In addition, *MALAT1 *knockdown reduced the cell migration rate. However, no effect on cell proliferation was observed [52].

***In vivo *studies using model objects**

The *in vivo *functions of *MALAT1 *have mainly been studied by xenotransplantation of human tumors or cell cultures into thymus-deficient mice. The *in vitro* studies in cell cultures and studies using tumor xenografts have revealed the contradictory effects of* MALAT1 *on tumor cell growth and invasion. Targeted inactivation of the *MALAT1 *gene in a breast cancer model in transgenic mice without altering the expression of neighboring genes was shown to promote lung metastasis, and this phenotype can be reversed by genetic insertion of *MALAT1*. Identically, *MALAT1 *knockout in human breast cancer cells confers metastatic ability, which is eliminated by *MALAT1 *re-expression [53]. Furthermore, MALAT1 stimulates mammary tumor growth: transfecting siMALAT1 into MDA-MB-231 and ZR-75-1 cell cultures suppressed the proliferation ability of cells, whereas subcutaneous injection of transfected tumor cells to mice also reduced tumor growth rate and size [48]. According to the results obtained for cell cultures (*MALAT1 *knockdown resulted in inhibition of the proliferation and invasion ability and triggered apoptosis in TNBC cell cultures), Jin et al. [45] subcutaneously injected *MALAT1 *knockout tumor xenografts to mice and obtained similar results: tumor growth was inhibited; tumor size decreased; MALAT1 hypoexpression triggered apoptosis of tumor cells and reduced their proliferation rate and the number of Ki-67-positive cells in the tumor. In a model of xenografts with siMALAT1, an influenced miR-124 inhibitor and miR-124+ inhibitor showed that MALAT1 overexpression is associated with CDK4 expression and cell proliferation, all controlled by the CDK4/E2F1 signaling pathway in breast cancer [25]. It is also worth noting that Yang et al. developed a mouse tumor xenograft model for detecting the *MALAT1 *function in HER2+ breast cancer: MALAT1 expression was significantly upregulated in HER2+ breast cancer both in cells and in tissues. *MALAT1 *silencing suppressed the proliferation of HER2+ breast cancer cells. The results seemed to suggest that MALAT1 could be a potential biomarker and a therapeutic target in HER2+ breast cancer [54].

Several studies have addressed the feasibility of targeting MALAT1 in order to improve the treatment of malignant neoplasms. Research into RNA therapy currently allows one to design RNA-based therapeutics, namely, antisense oligonucleotides (ASOs), which are small sequences complementary to mRNA carrying information about the protein under study, which can inhibit its synthesis [55]. Examination of the role of MALAT1 in breast cancer progression in the MMTV (mouse mammary tumor virus)-PyMT model showed that *MALAT1 *knockdown subcutaneously delivered ASO and reduced the metastasis rate. *MALAT1 *knockdown (20–80%) was achieved in mice injected with MALAT1-specific ASO1 or ASO_2_ compared to control mice that received the scrambled ASO (ScASO) control. The tumor growth rate was also reduced by 50% in mice in the experimental group compared to that in control mice injected with ScASO [56].

***In vivo *studies in breast cancer patients**

The published data suggest that MALAT1 utilizes different mechanisms for different molecular subtypes of breast cancer [2]. *MALAT1 *expression is upregulated in patients with TNBC, and those with elevated MALAT1 expression levels have a poor overall survival chance. Thus, Samir et al. investigated not only MALAT1 lncRNA, but also the X-inactive specific transcript (XIST). They successfully demonstrated that although miR-182-5p exhibited oncogenic activity, XIST had a preponderant effect on the regulation of the PD-L1 signaling pathway by inhibiting the oncogenic function of MALAT1 [57]. This fact can explain the findings obtained by Xiping et al. showing that MALAT1 suppression downregulates PD-L1 expression. This study demonstrated that MALAT1 gene editing can efficiently suppress the proliferation and invasion ability of triple negative and HER2+ breast cancer cells [2]. MALAT1 expression levels are much higher in TNBC samples than they are in HER2+ breast cancer samples. Lin et al. [32] showed that the downregulated or absent expression of MALAT1 is typical mostly of normal tissue, while MALAT1 overexpression is characteristic of breast, pancreatic, liver, lung, colorectal, and prostate cancers. MALAT1 mRNA expression proved also significantly upregulated in breast cancer tissues. These results are consistent with the findings made in earlier studies demonstrating that MALAT1 lncRNA can also promote cell proliferation and invasion in TNBC and lung cancer [57]. It follows from these data that MALAT1 can be used as a promising biomarker in the clinical diagnosis and prognosis of aggressive breast cancer tumors. In other words, it is clear that MALAT1 activation plays a crucial role in breast carcinogenesis. However, it is interesting to note that the serum levels of MALAT1 can also be a potential diagnostic oncomarker of breast cancer. In their *in vitro *study, Miao et al. showed that suppression of MALAT1 lncRNA significantly inhibited the proliferation, migration, and invasion of breast cancer cells, induced apoptosis and G1-phase cell cycle arrest, which has also been repeatedly shown in other independent studies. Furthermore, the serum level of MALAT1 in breast cancer patients was significantly higher than in patients having benign breast conditions (*p* < 0.001) [58].

On the other hand, when analyzing the RNA sequencing data (The Cancer Genome Atlas), Kim encountered the lowest MALAT1 expression levels in more aggressive tumors; MALAT1 expression in breast cancer cells was lower than that in normal tissue. This finding contradicted the results reported in other studies: in most cases, overexpression of the MALAT1 transcript in breast cancer cells compared to normal tissue was observed [25, 28, 45, 46, 59, 60, 61]. Kim et al. used the CRISPR-Cas9 genome editing tool to achieve *MALAT1 *knockout and observed an increased metastasis rate. Such differences in the results most probably had to do with the differences in the approaches used to obtain MALAT1 knockdown mice. Thus, according to the published data, MALAT1 overexpression is observed in the tumors of the ER+ and PR+ subtypes, as well as TNBC [27, 31, 46, 62]. Comparison of the expression levels of the transcript in TNBC and HER2-enriched breast cancer cells revealed MALAT1 overexpression in triplenegative cancer cells, which may be an indication that MALAT1 expression is correlated with the metastatic ability and that the differences are associated with the mediated participation of MALAT1 in different cellular processes [2]. MALAT1 overexpression is believed to be associated with poor tumor differentiation and resistance to hormone therapy [59, 62], while low expression might be associated with the relatively high five-year overall survival rate of breast cancer patients [63].

## CLINICAL SIGNIFICANCE OF MALAT1

Not only is *MALAT1 *usually overexpressed in different types of cancer, but it also frequently undergoes mutation. Some researchers have reported a high frequency of mutations in the *MALAT1 *locus (e.g., translocation in *MALAT1 *in renal cell carcinoma and gastroblastoma cells is established) [17]. Today, there are very few studies focusing on the association between* MALAT1 *mutations and breast cancer progression and the clinicopathological parameters of the tumor; so, it remains an open question whether this gene is a driver gene in breast carcinogenesis or not [64]. Kandoth et al. reported a low rate (1.1%) of *MALAT1 *mutations in breast cancer patients compared to other types of malignancies [31, 65]. However, the genome-wide association study of tumors collected from breast cancer patients conducted by Nik-Zainal et al. revealed a high rate of *MALAT1 *mutations (single nucleotide substitutions, insertions, and deletions), but it still remained unclear whether these mutations were driver mutations or resulted from the high tumor mutation burden in this genomic region [66].

*MALAT1 *was shown to belong to the group of genes of the luminal B breast cancer subtype:* MALAT1 *mutations are associated with such clinicopathologic parameters as a high tumor grade and high Ki-67 expression level. *MALAT1 *deletions and high frequency of insertion and deletion mutations (that most likely had arisen during transcription) were also observed in patients with the luminal subtypes of breast cancer [67]. This study also mentioned that *MALAT1 *mutations were unrelated to changes in gene expression levels; they probably had arisen during transcription as well [64]. The probability of activating the oncogenic effect of MALAT1 on cells can hardly be associated with gene amplification. This conclusion was drawn by Meseure et al., since the* MALAT1 *gene resides in the chromosome locus that is rarely amplified [31]. According to our data [68], the frequency of deletions in the locus where *MALAT1* resides in luminal B breast tumors amounts to 18%. Amplification at the 11q13.1 locus was observed in 10% of patients; in the vast majority of cases (72%), tumor cells had a normal copy number at this locus [68].

Furthermore, the MALAT1 lncRNA rs619586 polymorphism was shown to be associated with the response to platinum-based chemotherapy [69]. In the dominant genotypic model, the presence of the wildtype genotype (A/A) was found to be associated with a high chance of responding to chemotherapy by patients with non-small cell lung cancer (OR 0.60; 95% CI 0.36–0.97; *p *= 0.04), especially by patients younger than 57 years (OR 0.49; 95% CI 0.24–0.98; *p *= 0.04), males (OR 0.53; 95% CI 0.31–0.92; *p *= 0.02), smokers (OR 0.46; 95% CI 0.24–0.89; *p *= 0.02), and patients with squamous cell carcinoma of the lung (OR 0.24; 95% CI 0.10–0.60; *p* < 0.001) [69].

**MALAT1 as a prognostic factor**

According to the data reported previously, MALAT1 can be used as a promising biomarker in the clinical diagnosis and prognosis of aggressive breast cancer. Findings on the MALAT1 expression level can be a prognostic factor. An analysis of the data reported in 14 studies revealed that MALAT1 overexpression was associated with poor patient survival (HR = 1.95; 95% CI 1.57–2.41; *p* < 0.001) [48, 70, 71]. The low relapse-free survival rates associated with MALAT1 overexpression were also characteristic of patients with the ER-negative profile of tumor expression (HR = 2.83; 95% CI 1.02–7.83; *p *= 0.045) and for the group of patients having the luminal subtypes of breast cancer (ER+) and receiving tamoxifen therapy (HR = 2.56; 95% CI 1.04–6.0; *p *= 0.034) [62]. Similar results were obtained for patients with the TNBC and HER2+ subtypes of breast cancer having no lymphatic metastases; elevated MALAT1 levels correlated with a worse prognosis [27]. Elbasateeny et al. [72] arrived at a conclusion that not all TNBC patients have a poor prognosis; patients negative for one of the MALAT1 and BACH1, or both, have a satisfactory prognosis and so can be managed by breast oncoplastic conserving surgery. These data can explain the inconclusiveness of the findings obtained in independent studies. Later, Wang et al. conducted a meta-analysis, with special emphasis placed on metastatic spread, and showed that MALAT1 overexpression is associated with poor disease prognosis. The relapse-free survival of breast cancer patients with upregulated expression of this gene was lower in 95% of cases (HR = 1.97; 95% CI 1.25–3.09; *p *= 0.003), and no association between MALAT1 expression and lymphatic metastasis was detected (OR = 1.32; 95% CI 0.34–5.21) [73]. However, for the TNBC and HER2+ breast cancer samples, Xiping et al. revealed a positive correlation between the increased expression level of the MALAT1 transcript and the number of metastatic lymph nodes, as well as an inverse relationship between its expression level and the relapse-free survival rate of patients with the HER2+ subtype of breast cancer [2]. A different effect was reported for the metastasis-free survival rate of breast cancer patients: the decreased MALAT1 expression in these patients was associated with worse survival rates (HR = 0.81; 95% CI 0.67–0.99, *p *= 0.0420; HR = 0.65; 95% CI, *p *= 0.005) [23]. However, in this case, the conclusion was based on experimental results demonstrating that MALAT1 acts as an EMT inducer in breast cancer patients by activating the PI3K-Akt pathway. Therefore, there is no direct evidence of correlations between a low MALAT1 expression level and a worse prognosis.

In addition, a recent meta-analysis showed that high MALAT1 expression levels are associated with the PR+ tumor profile (95% CI 1.18–1.82; *p *= 0.0006) and, moreover, with decreased immune cell infiltration into the tumor, which may be one of the reasons for the poor survival prognosis in breast cancer patients with MALAT1 overexpression [71]. Finally, we would like to mention that Meseure et al. showed that both the expression level of the full-length MALAT1 transcript and the expression level of the alternatively spliced MALAT1 transcript (∆sv-MALAT1) carrying two deletions can be used as prognostic factors: ∆sv-MALAT1 hypoexpression in the tumor was observed in 19% of cases and was positively correlated with a large tumor size, ER-negative, PR-negative, triple-negative subtypes of breast cancer, and a poor metastasis-free survival chance [31]. Hence, it is fair to assume that alterations in gene expression affect the direction of tumor progression.

Importantly, MALAT1 is also a prognostic marker in human tumors of other localizations. Thus, according to the results of a study of prostate cancer cells resistant to enzalutamide (an antiandrogen used in prostate cancer treatment), the *MALAT1/AR-v7* axis (androgen receptor splice variant 7, AR) can be a promising therapeutic marker. The relationship between the expression of *AR-v7*, which contributes to the development of enzalutamide resistance, and the* MALAT1 *expression has been emphasized [74]. The expression levels of both genes in EnzR-PCa cells (the enzalutamide-resistant cell line) were higher than those in drug-susceptible cells. Administration of MALAT1 siRNA and/or ASC-J9 (5-hydroxy-1,7- bis(3,4-dimethoxyphenyl)-1,4,6-heptatrien-3-one) suppressed the progression of EnzR-PCa tumor cells. AR was shown to bind to androgen response elements (AREs) on the *MALAT1 *promoter. This interaction was inhibited in the presence of enzalutamide, thus boosting the activity of the *MALAT1 *promoter. In turn, MALAT1-siRNA inhibited *AR-v7 *expression [74].

Hence, the MALAT1 expression level can be used as a prognostic factor in breast cancer. The revealed patterns give grounds for inferring that MALAT1 lncRNA may indeed be a good predictive marker for selecting this treatment option.

## CONCLUSIONS

An analysis of the role of lncRNAs in the carcinogenesis of different types of tumors appears to be important, since new data on the principles of action of long non-coding RNAs would reveal the role played by the non-coding part of the genome in tumor pathogenesis, as well as supplement our knowledge about potential prognostic markers in cancer; breast cancer in particular. The metastasis-associated lung adenocarcinoma transcript 1 (MALAT1), which was recently discovered during a study of the mechanisms of metastasis in lung cancer, is of interest. This RNA is involved in numerous cellular processes such as transcription, splicing, metastatic spread, cell proliferation, etc. It can be inferred from a number of studies that a high level of this transcript is a marker of a poor survival likelihood for breast cancer patients and it can also be involved in the regulation of the mechanisms of EMT, invasion, and metastatic spread. For this reason, collecting data on this ncRNA is important in the search for more efficient methods to diagnose and treat malignant breast tumors. Further research into the functions of MALAT1 will allow one to understand the key mechanisms of tumor neoplasm initiation and progression.

## Abbreviations

snRNA: small nuclear RNA
BC: breast cancer
TNBC: triple-negative breast cancer
EMT: epithelial-mesenchymal transition
Δsv-MALAT1: small variant MALAT1
ER1: estrogen receptor 1
MALAT1: metastasis-associated lung adenocarcinoma transcript 1
mascRNA: MALAT1-associated small cytoplasmic RNA
MMTV-PyMT: mouse mammary tumor virus-polyomavirus middle T-antigen
ncRNA: non-coding RNA
sh-MALAT1: short hairpin RNA
siMALAT1: MALAT1 small interfering RNA
